# “I Don’t Want to Die on the Street”: Patient and Practitioner Perspectives on Street-Based Care for Older Adults Experiencing Unsheltered Homelessness

**DOI:** 10.1007/s11606-025-09591-7

**Published:** 2025-05-28

**Authors:** Anuva Mittal, Enya Lowe, Corinne Feldman, Alexis Coulourides Kogan

**Affiliations:** 1 USC Keck School of Medicine, Los Angeles, CA USA; 2 Department of Family Medicine and Geriatrics, USC Street Medicine, USC Keck School of Medicine, Alhambra, CA USA

**Keywords:** Aging, Older adults, Unsheltered homelessness, Serious illness, Ill-housed persons, Qualitative methods

## Abstract

**Background:**

Unsheltered older adults experiencing homelessness constitute the fastest-growing segment of the US unhoused population. Research shows that older unsheltered adults have more chronic health conditions and experience accelerated aging. Little is known about the unique considerations in providing care and support for this population.

**Objective:**

To explore the experiences of aging and managing serious illness from people experiencing unsheltered homelessness and from the street medicine team members who care for them.

**Design:**

Qualitative, semi-structured in-depth individual interviews.

**Participants:**

Street medicine team members and patients receiving street medicine in Los Angeles.

**Approach:**

Interviews were guided by a semi-structured interview guide developed by the team, audio-recorded, and transcribed verbatim. Field notes supplemented transcripts. Transcripts and field notes were analyzed by two independent coders following a thematic analysis approach rooted in grounded theory.

**Key Results:**

Eight street medicine team members with varying experience caring for patients experiencing homelessness (1–16 years) and from multidisciplinary backgrounds were interviewed. Team members were, on average, 39 years old (SD 7.4 years), 63% female, and 50% white. Eight patients were interviewed and identified as male (63%), having 3 + chronic health conditions (100%), and aged on average 56 years (range 50–71; SD 4.7 years). Thematic analysis of the interviews revealed two major themes on challenges and considerations for the following: (1) Caring for older adults experiencing unsheltered homelessness (subthemes: Medical, Interpersonal, Environmental, and Systemic), and (2) Shelter, long-term care, and end-of-life planning among older adults experiencing unsheltered homelessness (subthemes: Permanent shelter, Rehabilitative and institutional care, and End-of-life care planning).

**Conclusions:**

Team member and patient perspectives offered insight into significant challenges faced when trying to apply conventional healthcare practices to the unique circumstances of the unsheltered setting. Findings suggest actionable strategies with implications for both policy and practice to better meet the needs of unsheltered homeless older adults.

**Supplementary Information:**

The online version contains supplementary material available at 10.1007/s11606-025-09591-7.

## INTRODUCTION

The most recent nationwide data in the USA on homelessness reports 635,100 people experience homelessness on any given night with 40% experiencing unsheltered homelessness.^1^ Unsheltered homelessness means living in a place not designated for human habitation, including streets, vehicles, and parks. Though the number of adults experiencing unsheltered homelessness has been growing nationwide, California carries the greatest proportion with almost 49% of the nation’s unsheltered homeless population residing in the state.^1^ Furthermore, California’s unhoused population is steadily aging with a median age of 47, and the number of adults over age 50 experiencing homelessness is expected to double by 2030.^2,3^

This unsheltered, older population is growing and faces unique risks compared to housed individuals and younger people experiencing homelessness. Unhoused older adults are found to experience accelerated aging and mortality on the streets^2,4^ and face a greater number of physical, social, and emotional needs compared to their housed counterparts.^5^ On top of the health concerns older, unsheltered individuals face greater risks compared to those who are sheltered, including greater food insecurity, health comorbidities, and stress, and face higher rates of mortality when controlling for environmental and social factors.^6,7^ Few studies specifically studied the increased needs and vulnerabilities associated with the intersection of being both older and unsheltered.^4,8–10^ Given these statistics, finding solutions that specifically address the unique needs of unsheltered, older adults is imperative.

In response to the growing and alarming health crisis among people experiencing unsheltered homelessness, street medicine was developed as an alternative healthcare delivery model to better account for the unique circumstances of this population and better meet their healthcare needs.^11^ Sometimes called “backpack medicine” because of the way it is delivered, street medicine is a unique model of full-spectrum primary care^12^ delivered on the street directly to people experiencing unsheltered homelessness via walking rounds. As opposed to traditional four-walled medical settings or mobile clinics/vans, going to a patient's encampment flips the patient-provider power dynamic and reduces a multitude of person- and system-level barriers to care commonly experienced by people experiencing unsheltered homeless such as lack of transportation, concern with leaving belonging unattended, competing priorities for survival, past trauma, interpersonal concerns, fear of re-traumatization, and fear of discrimination, among others.

As street medicine programs proliferate across the country, an opportunity exists to better meet the needs of people experiencing unsheltered homelessness, in general, and older unsheltered adults, specifically. Additionally, trends now show a national move toward codifying street medicine in insurance coverage, encouraging clinicians nationwide to create better systems for taking care of unhoused individuals. In October 2023, the Centers for Medicare and Medicaid Services initiated reimbursement for clinicians who provide care to unsheltered individuals in a “non-permanent location on the street or found environment, not described by any other POS code.”^13^ Therefore, eliciting team member and patient experiences with street-based care is timely and applicable in the current landscape of medicine. The study purpose was to explore the experiences of aging and managing serious illness from people experiencing unsheltered homelessness and street medicine team members to offer future directions for healthcare and policy development.

## METHODS

We conducted one-time, qualitative interviews with street medicine patients and team members based in Los Angeles to learn about the experience of aging and managing serious illness while also experiencing unsheltered homelessness. The interview guides included open-ended questions about patients’ and team members experiences with management of serious illness. The institutional review board at the University of Southern California approved this study as exempt research, requiring verbal consent and study information sheet, only; no written consent declarations (Approval Number UP-20–00853). The Consolidated Criteria for Reporting Qualitative Studies Guidelines (COREQ) informed this study report (see Appendix A).^14^

### Setting

This study was conducted with USC Street Medicine: one of the first US-based street medicine programs sponsored by an academic medical center. USC Street Medicine provides comprehensive, full-spectrum primary care and social services delivered directly to people experiencing unsheltered homelessness in their own environment by interdisciplinary teams serving various geographic areas in Los Angeles. Core team members include a prescriber (physician, physician assistant, or nurse practitioner), registered nurse, social worker, and a community health worker with lived homeless experience. Ancillary members who circulate between the teams include: psychiatrist, physician trained in point-of-care ultrasound, HIV-specialist, family medicine residents, occupational therapy residents, medical students, and physician assistant students. In fiscal year 2023–2024, the program provided care to approximately 2000 unique patients (40% aged 50 +) and conducted over 11,000 patient visits.

### Procedures and Participants

We used a convenience sampling approach for interviews. A female research team member (EL) with a degree in anthropology, formal training in qualitative methods, and no prior or existing relationship with potential participants accompanied the street medicine team on clinical rounds for 3 days to invite patients to voluntarily participate in an interview at the conclusion of their usual medical encounter. Potential participants were given an explanation of the study, interview procedures, and a physical copy of the study information sheet. Nine people were approached with eight agreeing to be interviewed; the person who refused the interview also did not engage with the street medicine team in their medical visit. Individuals agreeing to participate were interviewed on-the-spot by the same researcher (EL). Interviews were led by a six-item semi-structured interview guide developed by all members of the study team and reviewed for appropriateness and face validity by a street medicine team member with previous lived experience with homelessness. Interviews were audio-recorded with participant consent and transcribed verbatim for analysis. On average, interviews lasted 31 min (SD = 16 min; min: 10.25; max: 55.26). Field notes were also collected by the interviewer to supplement recordings performed in a noisy location, to avoid potential recall bias by the interviewer, and to add details on environmental and contextual factors (i.e., weather conditions, conditions of place of interview/encampment, participant demeanor, etc.). Participants were given snacks and personal hygiene items valued at seven dollars. This was based on community advisory board input to not offer direct monetary compensation to avoid participation coercion.

After completing patient interviews, we used a multidisciplinary convenience sample at USC Street Medicine to invite participants for an individual interview with study personnel (ACK and/or AM) via HIPAA-compliant web conferencing (Zoom). Interviewers did not have a prior relationship with interviewees. An IRB-approved email invitation drafted by the study principal investigator (ACK) was sent to the program listserv (25 people) by a senior leader on the street medicine team and study team (CF). Interested individuals replied to the email to schedule their interview and address any questions. Interviews were guided by a 6-item semi-structured interview guide developed by all members of the study team and reviewed for appropriateness and face validity by a palliative care physician (KL) at an outside institution. Interviews were audio-recorded and transcribed verbatim by a professional transcription service. On average, interviews lasted 44 min (SD = 11 min; min: 23; max: 56). No member checking occurred as interview transcripts were not provided to any participant.

### Analysis

To identify and code the themes that emerged from the data, the two-person female coding team (ACK and AM), both with formal graduate-level training in qualitative coding methods (PhD in Gerontology and MS in Narrative Medicine, respectively) used a Thematic Analysis approach that incorporated elements of Grounded Theory; an explorative and inductive method of data analysis and construction.^15^ Though not intending to create a new theory, the coders appreciated the iterative coding process outlined in Grounded Theory. Excel was used to note initial codes from the transcripts and field notes with an open coding method, an “analytic process through which concepts are identified and their properties and dimensions are discovered in data,” to first independently identify pertinent topics that emerged from the transcripts.^16,17^ Research team members individually grouped the resulting codes into overarching categories in the process of axial coding. Next, emerging theme discussion, relationship identification, and code consolidation occurred.

The two coders first met after coding all of the patient interviews and the first two team member interviews. They had completed the open and axial coding tasks individually beforehand. After developing an initial joint set of themes, they coded the next four team member interviews individually, drawing upon the iterative coding process outlined in Grounded Theory. The iterative process brought greater cohesion throughout the analysis by reflecting on previously devised themes while coding new transcripts. After the next four team member interviews were coded and discussed between the two coders, there was agreement that saturation of themes was reached, as new concepts and themes were no longer found. However, the remaining two interviews were conducted to achieve multidisciplinary representation from team members and because the additional team members had already volunteered to participate and were scheduled to be interviewed. To ensure objectivity, an adjudicator (EL) reviewed the initial set of themes alongside the transcripts to ensure that no salient ideas were overlooked.

Lastly, working independently once more, the two primary coders (ACK and AM) re-applied and reviewed the final set of thematic categories to all the transcripts to ensure that the participants’ voices were adequately reflected in the final results. During this second independent coding process, relevant direct quotations for each theme were also noted to support concepts. Finally, they came together again and reviewed the themes and quotes, further paring down, sorting, and consolidating until 100% consensus was reached.

## RESULTS

### Participant Demographics

Eight street medicine team members and eight patients were interviewed (*N* = 16). The average age of the clinicians was 39 years (SD 7.3 years), and 63% were female while 50% were white. They had varying years of experience in street medicine (mean 1.5 years). Participants were multidisciplinary and included roles such as medicine (MD and PA), nursing (RN), community health worker (CHW), and occupational therapy (OT). Patients most commonly identified as male (63%), having 3 + chronic health conditions (100%), and aged on average 56 years (SD 4.7 years) (see Table 1).

**Table 1:** Characteristics of Patient and Street Medicine Team Member Sample. Eight Street Medicine Clinicians and Eight Patients Were Interviewed (*N* = 16). More Robust Patient Data Was Not Collected due to Concern for Stigma Among Patients

	Team members	Patients
	(*n* = 8)	(*n* = 8)
Age, years (mean ± SD)	39.4 ± 7.3	56 ± 4.7
Gender identity		
Female	50%	37%
Male	38%	63%
Non-binary	12%	-
Racial identity		
Hispanic/Latino/a	25%	-
Ethnic identity		
Native American	12%	-
Asian	25%	-
White	50%	-
Black	0%	
Other	12%	-
Team members’ disciplines		-
MD/DO	25%	
PA	37.5%	-
RN	12.5%	-
OT	12.5%	-
CHW	12.5%	-
Experience in street medicine at USC		
Mean ± SD	1.5 years ± 1.7	-
Min–max	1 month–4 years	-
Experience in caring for patients experiencing homelessness		
Mean ± SD	10.4 years ± 4.6	-
Min–max	1 year–16 years	-

### Qualitative Themes

Inductive analysis of the interviews revealed two major thematic categories with multiple subthemes: (1) Challenges and considerations for care of older adults experiencing unsheltered homelessness, and (2) Challenges and considerations for shelter, long-term care, and end-of-life planning among older adults experiencing unsheltered homelessness. Select pertinent quotes were included in the body of the text but additional quotes can be found in Table 2.

1. Challenges and considerations for care of older adults experiencing unsheltered homelessness

**Table 2:** Perceptions of aging and managing serious illness by patients receiving street medicine and street medicine team members (N=16)

1. Challenges with caring for older adult experiencing unsheltered homelessness	
1a. Medical	
OT	“There [do] sadly tend to be more comorbidities [for older adults on the streets], of course–musculoskeletal changes that keep people less active…Things add up–heart attacks, strokes, everything sort of escalates. I would say there are probably higher rates of morbidity…and, therefore, more challenges to treat it.”
MD-1	“There are specific kinds of medications that are incredibly difficult…impossible if you are living on the streets [like] insulin, a good example of that needing refrigeration.”
Patient 1	“Sometimes [certain medications] make you tired and then what do you do? Or sometimes you can’t be in the sun [due to a certain medication] and then what do you do? And they tell you, ‘Oh, go hang out at the mall for a quick [air conditioning] fix,’ like that’s so easy to do.”
1b. Interpersonal	
MD-2	“The older folks, not all, but many of them do seem to be more on their own. When people are on their own, there’s a lot of isolation because nobody else is talking to them, and that’s often really hard for them.”
PA-1	“I think there’s the typical things that happen to people when they’re inside [that] continue to happen when you’re outside as an older adult. But then you also have this incredible potential for exploitation because you are vulnerable as a result of some of those normal aging processes, but then there is sort of an ageism on the street. If you're older, then you’re less likely to be able to defend yourself or maybe to be more dependent on other people because you can’t walk as far or whatever those cases are.”
1c. Environmental	
Patient 2	“The environment is really harsh, so it could be a little rough at times.”
1 d. Systemic	
RN	“I can’t imagine the hundreds and thousands of older adults out there, who don’t have Medicare Part D… and don’t have the access to someone like the street medicine team or even the homeless service provider team or a social service provider. I can’t imagine that.”
CHW	“[Patients on the street] tend to maybe lose [medications] more or get [them] stolen more, so a lot of times what we like to do on the streets is give them a pill box [for] a week, two weeks…because if they continue to get them stolen or lost then we can’t refill them.”
MD-1	“It takes more time and a little more TLC to gain patients’ trust…In more traditional settings of healthcare, they have been stigmatized and treated poorly, so we’re coming in with a model trying to counter that narrative.”
PA-1	“When caring for individuals on the street, we should not ever [let] our relationships with our patients become purely transactional, because to the patients, it is much more than that.”
2. Challenges with shelter, long-term care, and end-of-life planning	
2a. Permanent shelter	
PA-2	“Getting him into the PRK site was great because he was in a room and had a bed. But his community that was around him [while living outside was] giving him food and giving him cigarettes and giving him alcohol. They weren’t there anymore because he was now in this Project Roomkey site. He was warm, and he had a bed, and he probably wouldn’t die overnight, but we were worried about him going into withdrawal from alcohol as well as [being] able to just function on his own.”
PA-2	“It would be cool to have a case manager on our team specifically to kind of manage some of that stuff [housing] where it’s all under one person or something…It’d be cool to have some more people like that on our team.”
2b. Rehabilitative and institutional care	
PA-3	“Having a real housing option would be great and just having more services available. Often, what a lot of patients need is some sort of recuperative care place where they can go because they have all these needs, and that’s been a big issue for patients who don’t necessarily belong in the hospital and then can’t necessarily get into a skilled nursing facility. We’ve struggled to find proper placement for a lot of our patients who are older.”
MD-2	“There are other settings that would also sometimes be more appropriate like…[a] nursing home or recuperative care. But the way the system is set up, it’s really designed for those placements to happen at hospital discharge, not from an outpatient setting.”
2c. End of life care planning	
Patient 1	“Recently, a friend just passed away, and she had cancer, and when she found out she was already Stage 4. They only gave her 10 days to live. So that was like, ‘whoa.’ It was so sad because to know that you only have less than 10 days, that is so–the things that we take for granted in life like I’m too young, I have five kids, and that’s a pretty scary thing because I can’t wait to be a grandma, and I might not ever get to see those [grandchildren].”
PA-2	“I would really like to start doing [conversations about advanced directives] more. It’s just I feel like there’s no time to have [them]…I know that’s a very important conversation…I need to make time for it, and it’s really challenging, and we don’t have a lot of time because I don’t want to rush that kind of thing.”
Field notes	“She said she knew a friend that recently got sick. The friend got pneumonia and she tried to call an ambulance but one never came. The friend passed away.”
MD-2	“That’s some heavy information, to be someone’s secret keeper, or be the only person that knows what that person’s wishes were or to carry out.”
PA-1	“When you’re asking [about advance care planning and end-of-life wishes] in the hospital or when you’re asking in the nursing home or whatever, I never felt like I was going to be the one who actually had to carry out those wishes or that I was the one that would have to be like, ‘Hey, they wanted a Catholic burial.’ Somebody else always knew that information.”

The challenges described by participants with caring for older unhoused adults were around four subthemes: medical, interpersonal, environmental, and systemic. Clinicians described difficulties around reframing care to fit patients’ environmental needs and provided specific examples of challenges resulting from inflexible healthcare systems and financing policies that do not account for the unique circumstances associated with unsheltered homelessness.

- 1a. Medical

Clinicians described difficulties with managing patients’ many diagnoses and adapting prescription medications to patients’ living conditions. A prominent factor contributing to these challenges was the absence of or lack of clarity on patients’ prior medical histories. One clinician explained that, “…having a diagnosis [is] a privilege.” Meaning, access to consistent healthcare, where diagnostic tests are performed, and diagnoses could be made and addressed, was a privilege that not everyone has. Without information on previous health records, clinicians faced difficulty in disentangling potential long-term health issues that could affect the patient’s current medical presentation. One clinician described these encounters as opening “Pandora’s box:”

It’s a little bit [like] opening Pandora’s box…We recently had a patient, who had not seen any kind of primary care…He thought he had no diagnoses but, in fact, he had a *lot* of things going on and eventually ended up dying of metastatic lung cancer; things that were preventative or at least treatable.—PA-1

After making diagnoses, clinicians’ next challenge was determining which medications to prescribe. Some commonly prescribed medications were not conducive to the living conditions of people experiencing unsheltered homelessness with food and water insecurity. For example, one physician said:

For high blood pressure, usually the first line is diuretics. However, in the street, the access to water has to be factored in. A lot of individuals may not often get Lasix or spironolactone or HCTZ because of the fact that they may over dehydrate.—RN

Team members and patients alike emphasized the importance of learning about patients’ unique living conditions and adapting treatment recommendations to their specific environments. Factors such as hydration, nutrition, reliable access to a toilet, drowsiness, access to refrigeration, and reprieve from the weather were all described as important considerations during clinical decision-making.

- 1b. Interpersonal

At the interpersonal level, clinicians identified that older unhoused adults often faced more social isolation due to a lack of connection with family, complicated relationships with street-based friends, and age-based targeting (i.e., agism). One team member explained:

If [older unhoused individuals] are on social security, they’ll get their card stolen, or [others on the streets will] make them take their money out and give it to them. They’re really vulnerable on the streets, and that’s probably more of what really keeps us going back and trying to help as much as possible, because again, they’re just super vulnerable.—CHW

Even when patients on the streets had other community members around them that they could talk to, team members explained that these acquaintances were not necessarily supportive of the patients’ health goals. For example:

They would come around [an older patient] and just hang out with him during the day, which I think he misses a lot. I think he feels really lonely. But sometimes they’re not the best influences on them.—PA-2

Both the incidence and quality of interpersonal relationships were discussed only by street team members, not patient participants.

- 1c. Environmental

Patients and clinicians both described difficult environmental circumstances that were captured in field notes:

[Interview conducted] behind a gas station near an offramp [from the freeway], tent is located under an overpass, a lot of noise.—Field notes

These conditions often forced older unsheltered patients to choose between essential needs, such as safety, and accessing and following up with medical care. One clinician said:

If you’re significantly ill on the street, you have the most unbelievable set of competing priorities…Am I going to eat? Do I have water? Can I find some place to go to the bathroom?…Maslow’s hierarchy of needs are not a given on any one day.—PA-1

Clinicians also described the way that seemingly minor illnesses could snowball into more serious complaints in the midst of the “competing priorities” that patients had to balance on the streets:

I worry that things that are easily preventable or treatable in a different setting will end them up in the hospital…[Ensuring belongings are safe will] take priority over, ‘Oh, it kind of burns when I pee.’ But that can become a full-blown septic picture easily.—MD-1

- 1 d. Systemic

At the systems-level, patients’ insurance coverage often prevented them from receiving necessary therapies while living on the streets. Many patients were not familiar with their insurance coverage or lacked the resources and documentation to connect with their insurance provider. One patient explained:

I had this whole issue with my birth date, so I went to a clinic trying to get help with my knee. They said, ‘You’re not in the system. You don’t even exist.’ I didn’t have access to help for quite a while because of this issue with my birth date…I just started trying to do homeopathic stuff. It seemed like it was an infection and then all of a sudden, it was an *infection*.—Patient 2

Additionally, team members and patients discussed medication loss and theft. Systems-level rules around refilling medications before 30-days of initiation or last fill complicated patients’ abilities to sustain treatment. Furthermore, team members and patients acknowledged the systemic stigma that unsheltered patients face when engaging with traditional, four-walled medical institutions. One clinician said:

Unhoused folks– a lot of times they feel like they go, and they’re not treated well. They’re not treated fairly…we hear this all the time.—MD-1

2. Challenges and considerations for shelter, long-term care, and end-of-life planning among older adults experiencing unsheltered homelessness.

2a. Permanent shelter

Street medicine patients and team members offered recommendations for improved support for older adults experiencing unsheltered homelessness. After giving the caveat that a one-size-fits-all approach would not serve every unhoused individual, team members identified resources that could help their older patients. A physician assistant stated:

Many [unsheltered individuals] are going to need permanent, supportive housing where they’ve got those wraparound services that they can engage routinely with [like] case management services to help maximize their Medicare benefits, all of those kinds of things that they are very unlikely to navigate on their own without any support.—PA-1

Team members and patients also acknowledged that shelter alone was not always sufficient for patients especially if they relied on their community for daily care. Interviewees advocated for social avenues in sheltered settings:

Well, emotionally, I realized that being immobile out [on the street] is just not an option. That would be terrible…To be honest with you, as far as being in a house, I think it’d be more depressing. You’re stuck in a little cubicle and having this around you.—Patient 2

Based on clinicians’ experiences, ancillary services to replicate the social support that patients received on the streets would be an important facet of an effective sheltering system.

2b. Rehabilitative and institutional care

Aside from advocating for more permanent housing solutions, street medicine team members also described difficulties with connecting unsheltered older adult patients to temporary housing services. Clinicians explained that some of their older patients, who were not ready for permanent housing, would occasionally need temporary respite to manage exacerbations in their medical conditions. However, their medical concerns would not be dire enough to warrant a hospital admission or skilled nursing facility placement, leaving them to manage their conditions on the street. In particular, clinicians mentioned that current temporary sheltering options were inadequate due to a lack of healthcare resources at these sites for older adults with medical comorbidities:

Our emergency sheltering systems are not designed for older adults…[Shelters] get scared off by medical complexity. If they feel like someone is too medically complicated for them, they won’t allow them to stay in the shelter.—PA-1

2c. End-of-life care planning

Both street medicine patients and clinicians discussed death and dying. Patients discussed that seeing people they knew on the streets die or receive life-altering prognoses made them feel apprehensive and feel a lack of control about their own future. One patient said:

I don’t want to die on the street.—Patient 2

Patients also expressed concern about the perceived inevitability of death and their need to communicate their wishes around handling their bodily remains and possessions. Clinicians echoed this sentiment by emphasizing the value of receiving a diagnosis that would help patients exercise control over their remaining life trajectory:

It’s a luxury to know that you might not survive a diagnosis that you have. You had the luxury of having a diagnosis and having a prognosis and maybe being able to decide what to do in that time…I think any time [patients] have a heads up…if there are things that they wish to do, wish to reconcile, wish to get right with, they have that opportunity.—PA-1

Street medicine team members discussed the differences between street-based and hospital-based end-of-life conversations. Clinicians explained how, in the hospital, they rarely had the responsibility of carrying out the logistics of their patients’ end-of-life wishes whereas this was more commonly asked of clinicians in the context of street medicine. Clinicians described how “heavy” they felt being the “secret keeper” for their patients in these situations. As a result, they discussed needing both more time and a standardized way to document patients’ end-of-life wishes so that they could be more broadly known and honored by the street medicine program when needed.

However, even if street medicine clinicians wanted to carry out hospice care for their patients, they noted difficulties in establishing this care due to patients’ lack of physical address and potential environmental concerns, such as an open fire or other heating and cooking sources:

One of the main issues though with hospice was that he needed oxygen. And there’s a lot of problems with that. As you could imagine, there’s no electricity, but also he cooked over an open fire. And so we couldn’t find a durable medical equipment company that would say, ‘Sure, we’ll bring an oxygen tank next to the side of the highway where you cook over an open flame,’ in a residential neighborhood, by the way.—PA-1

## DISCUSSION

The purpose of this study was to descriptively explore the experiences of aging and managing serious illness from people experiencing unsheltered homelessness and from the street medicine team members who care for them. Results shed light on the unique variables and circumstances to consider in caring for unsheltered older adults. Our study builds upon existing studies on the experiences of unsheltered older individuals and confirms the elevated health needs they face.^4,8,9^ Further, our study uniquely offers the experience of street-based team members, which has not been documented extensively. Healthcare professionals are typically trained in and have work experience in traditional four-walled settings, which is very different from street-based care. As formal street medicine workforce development programs are developed,^18^ it is important to consider the special needs of the aging unsheltered population.

In the context of challenges with medical care for unhoused patients, prior studies similarly demonstrate a high disease burden among older unhoused individuals with a prevalence of both physical and mental symptoms.^19^ Furthermore, firsthand interviews with unhoused individuals show similar difficulties with storing medications or having them stolen.^20^ Given the rising number of unhoused older individuals and the fact that they have greater comorbidities compared to their younger counterparts,^21^ creating lasting solutions to specifically address their healthcare and social needs becomes more imperative.

Distinguishing between people experiencing unsheltered and sheltered homelessness is imperative in the design and provision of medical care and in research. This study focused on people experiencing unsheltered homelessness, and results highlight the unique challenges for basic survival that often impede a person’s ability to seek traditional medical care. For example, participants in this study described barriers related to “competing priorities” of daily necessities and medical care in unhoused individuals’ lives; an idea that was introduced as early as 1997 when characterizing the unhoused population’s needs.^22^ Similar ideas have been brought up in other vulnerable populations, including previously incarcerated individuals and women diagnosed with HIV.^20,23,24^ Creating housing options that take care of patients’ everyday needs, including providing safe storage of food and water and a place to store personal belongings, will allow this population to divert their attention to their medical needs. However, as seen in this study and others, current permanent shelter options are inappropriate for the medical and mobility needs of older unhoused adults.^25^ Clinicians in our study advocated for “wraparound services,” or a centralized, interdisciplinary team of social workers and case managers that could follow patients. This system would reduce some of the burden from clinicians, which other studies have also recommended.^26–28^

In terms of temporary housing structures, other studies have similarly found that subacute facilities refuse patients experiencing homelessness due to stigmas related to substance use and mental health.^29^ Temporary housing structures include those between hospital discharge and community reintegration, such as skilled nursing facilities that can provide intermediate wound care that would be too dangerous to manage on the streets. Studies have found that older unhoused individuals have higher rates of initial emergency department (ED) visits and repeat ED visits compared to their housed counterparts.^30,31^ Additionally, discharge to temporary rehabilitation facilities or longer-term living facilities has been found to be associated with lower risk of readmission of unhoused individuals.^32^ Creating sustainable temporary housing structures and medical respite for unhoused individuals will not only help them with their health needs after hospital admission but also take the pressure of difficult discharges off hospitals.

Lastly, another finding in this study detailed the unique variables clinicians faced when initiating conversations about end-of-life care. They recommended more robust training for carrying out these conversations. Research on end-of-life conversations among unhoused patients offers valuable insight into patients’ perspectives, elucidating their concerns about the uncertainty of dying without having close friends or family nearby or not knowing how or where they will be buried.^33–37^ Our study found that clinicians worry about properly honoring their patient’s end-of-life wishes in the face of time constraints and poor preparation on counseling unsheltered patients on this topic; similar to what others have found.^37,38^ Given the unique factors to consider on the street with end-of-life care, creating resources that include tailored topics and conversation starters may benefit street medicine team members and patients alike. This work is already underway among formerly unhoused older adults living in permanent supportive housing.^39^ An important direction for future research would be translating and modifying this work for an unsheltered, street-based population.

### Limitations

While this study adds to the growing field of understanding street-based healthcare and aging among people experiencing unsheltered homelessness, findings may be limited. First, the data were collected from a single institution. Secondly, the sample size for both patients and team members was relatively low. However, the purpose of exploratory qualitative research is not generalizability but instead insight into specific populations’ concerns. Engaging in team reflexivity is a critical step in the data validation process and while we took numerous steps to mitigate the impact, it is possible that study team members’ social position may have impacted interactions with study participants, data interpretation, and knowledge production. Despite the potentially limiting factors, this study recruited a diverse, multidisciplinary sample of street medicine team members. Though the study is unique to California, the state contains 49% of the nation’s unsheltered older population with 68% of California’s overall unhoused population being unsheltered,^1^ still making it relevant to a sizable population. Lastly, more robust patient demographic data could have contributed contextual details to this study, including allowing for analyses into the interplay between multiple marginalized identities (i.e., gender, race and ethnicity, etc.) and unsheltered homelessness. However, interviews were conducted anonymously with minimal demographic data collected to reduce participant stigma.

## CONCLUSION

This study captures the unique perspectives of street-based team members and the aging unsheltered homeless population: the fastest growing subgroup of the unhoused population across the USA. With input from both care recipients and team members, this study describes the firsthand challenges in short- and long-term management of care, shelter, and end-of-life planning for this population. Future program efforts should focus on developing innovative models of shelter and housing for older adults experiencing unsheltered homelessness and research should evaluate these models. Additionally, developing and evaluating street-based models of geriatric care and advanced care planning are warranted. Policies should be reevaluated to include special considerations for older unsheltered adults, including expedited pathways to housing and supportive services.

## Supplementary Information

Below is the link to the electronic supplementary material.

Sumpplementary file 1: COREQ checklist (PDF 653 KB)

Supplementary file 2: Team member question guide (DOCX 18 KB)

Supplementary file 3: Patient question guide (DOCX 20 KB)
