# Supplementary material for: “I Don’t Want to Die on the Street”: Patient and Practitioner Perspectives on Street-Based Care for Older Adults Experiencing Unsheltered Homelessness
Source: J Gen Intern Med. 2025 May 28;40(13):3003–12. doi: 10.1007/s11606-025-09591-7 (PMC12508372; doi:10.1007/s11606-025-09591-7)
Supplement: Supplementary file 2 — Team member question guide (DOCX 18 KB) [file 11606_2025_9591_MOESM2_ESM.docx]

**Supplementary Material:**

**Street Medicine Team Member Interview Protocol**

1. **What is it like caring for older adults and people with serious illness that live outside?**

-What is different about caring for older adults than other patients?

-What is different about caring for seriously ill patients than other patients?

-What barriers do you experience in caring for these patients?

-What are the particular challenges around managing medications for older adult and seriously ill patients?

1. **In your experiences and observations, how have your patient’s friends and/or family members been involved in the care and support of older adult or seriously ill patients that live outside?**

(*Probe*: caregiver support, community, family involvement)

1. **What would help you and the team better care for older adults and people with serious illness that live outside?**
2. **What “keeps you up at night” about caring for older adults and people with serious illness that live outside?**

-What do you worry about for these patients?

-What can we do to address your concerns/worries?

*Now I’d like to switch gears a bit and ask about violence and victimization among older adults that are experiencing homelessness*:

1. **What has been your experience with older adult patients being victims of violence?**

-What do you think could help homeless older adults at risk for violence?

- What puts someone at greater risk of victimization?

1. **Have you ever had to file an APS report for one of your patients?**

-How did that go?
