# Supplementary material for: “I Don’t Want to Die on the Street”: Patient and Practitioner Perspectives on Street-Based Care for Older Adults Experiencing Unsheltered Homelessness
Source: J Gen Intern Med. 2025 May 28;40(13):3003–12. doi: 10.1007/s11606-025-09591-7 (PMC12508372; doi:10.1007/s11606-025-09591-7)
Supplement: Supplementary file 3 — Patient question guide (DOCX 20 KB) [file 11606_2025_9591_MOESM3_ESM.docx]

**Supplementary Material:**

**Patient Interview Protocol**

**Background/Context**

1. **Thinking about our team that comes to see you and talk about your health, how has that been for you?**
   1. What have you liked about getting visits from our team?
   2. What have you *not* liked about getting visits from our team?
   3. Is there something more they can do that would be helpful to you?
   4. What is important to you when people talk to you about your health?
   5. How does it feel to have students involved in your care here?
   6. What do you think students need to know about homelessness in order to care for you and others in a similar situation?
2. Has your experience with Street Medicine been different than other types of medical visits you have had in the past? *(How does SM compare to “usual” medical care? Which do you prefer?)*

**Illness and COVID**

1. **Have you ever known anyone or had a friend on the street that was seriously ill or sick?**
   1. ****If ‘yes’***:
      1. Do you know what they were sick with?/died from?
      2. Did they get any help when they needed it? From whom/where?
      3. How did you feel about their situation?
2. **What is it like to be sick when you live outside?**
   1. What about when sickness drags on?
   2. What is it like to manage medications when you live outside?
      1. Challenges/storage/access/refills/opioids/police/others
3. **What is hardest about getting sick or feeling sick when you are living outside?**
   1. What do you need the most during those times? *(Probe: What kind of help does a sick person need when living outside?)*
      1. What problems do you face getting that?
   2. What is the role of friends during times when you are sick?

*(Probe: How do friends help each other on the street?)*

- 1. What is the role of family during times when you are sick?
  2. Do you have someone in your life that could speak for you and make decisions if you were unable to do that for yourself?
     1. If ‘yes’: have you talked to them about your wishes and what you would want or not want for yourself? Have you put your wishes in writing?/advance directive?
     2. If ‘no’: Have you put your wishes in writing?/advance directive?

1. **What matters most to you right now?**
   1. Is there anything that is bothering you now? Since you got here?
